# Defining the Pluripotent Marker Genes for Identification of Teleost Fish Cell Pluripotency During Reprogramming

**DOI:** 10.3389/fgene.2022.819682

**Published:** 2022-02-11

**Authors:** Huajin Li, Wenting Xu, Sijia Xiang, Leiting Tao, Wen Fu, Jinhui Liu, Wenbin Liu, Yamei Xiao, Liangyue Peng

**Affiliations:** ^1^ State Key Laboratory of Developmental Biology of Freshwater Fish, Hunan Normal University, Changsha, China; ^2^ School of Life Sciences, Hunan Normal University, Changsha, China

**Keywords:** fish, pluripotency, marker gene, stem cell, IPS (induced pluripotent stem) cell

## Abstract

Pluripotency is a transient state in early embryos, which is regulated by an interconnected network of pluripotency-related genes. The pluripotent state itself seems to be highly dynamic, which leads to significant differences in the description of induced pluripotent stem cells from different species at the molecular level. With the application of cell reprogramming technology in fish, the establishment of a set of molecular standards for defining pluripotency will be important for the research and potential application of induced pluripotent stem cells in fish. In this study, by BLAST search and expression pattern analysis, we screen out four pluripotent genes (*Oct4*, *Nanog, Tdgf1,* and *Gdf3*) in zebrafish (*Danio rerio*) and crucian carp (*Carassius*). These genes were highly expressed in the short period of early embryonic development, but significantly down-regulated after differentiation. Moreover, three genes (*Oct4*, *Nanog* and *Tdgf1*) have been verified that are suitable for identifying the pluripotency of induced pluripotent stem cells in zebrafish and crucian carp. Our study expands the understanding of the pluripotent markers of induced pluripotent stem cells in fish.

## Introduction

Pluripotency is defined as the potential of specific cells which can differentiate to cells from three germ layers under certain inducing conditions (Hanna et al., 2010). The embryonic stem (ES) cells are originated from early embryos, and possess the capabilities to differentiate into various cell and tissue types desired (Hoffman and Merrill, 2007; Yi et al., 2010; Wang et al., 2011). In mammals, traditional ES cells are derived from the blastocyst inner cell mass (Evans and Kaufman, 1981; Notarianni et al., 1990; Thomson et al., 1995; Thomson et al., 1998). The state of pluripotency is controlled by highly interconnected pluripotent gene regulatory networks (Ng and Surani, 2011; Li and Belmonte, 2017). Series of pluripotent markers have been reported in mammalian, most of which are transcription factors (Surani et al., 2007; Li and Belmonte, 2017). These transcription factors are generally expressed in the early stages of embryonic development, but significantly decreased in most differentiated tissues (Thiagarajan et al., 2014; Li et al., 2020). They interact with a variety of protein complexes to regulate the expression of multiple genes and maintain the pluripotency and self-renewal ability of ES cells (Paranjpe and Veenstra, 2015). However, studies showed that there were differences in the types of genes involved in maintaining cellular pluripotency for different species (Ralston and Rossant, 2010; Robles et al., 2011). The stage-specific embryonic antigen 1 (SSEA1) was regarded as a pluripotency marker in mouse cells, but in humans, it was a differentiation cell marker (Shamblott et al., 1998; Brambrink et al., 2008).

The research on fish pluripotent stem cells begins with the culture of ES cells (Hong et al., 1996, 1998; Barnes et al., 2008; Yi et al., 2010; Li et al., 2011; Robles et al., 2011; Wang et al., 2011; Ho et al., 2014; Hong et al., 2014; Fan et al., 2017). Xiao and his team have shown that zebrafish embryonic cells experienced a very short pluripotent state from zygotic genome activation to the oblong stage (Xiao et al., 2016). The blastocyst stage of embryos is also considered to be the most suitable period for the culture of fish ES cells (Christen et al., 2010). Up to now, some multiple pluripotent factors and related coregulators have been reported in fish species (Robles et al., 2011; Wang et al., 2011; Kumar et al., 2020). There found some differences in pluripotent markers between fish and mammals. Likewise, *sox2* was one of the key mammalian pluripotency genes (Surani et al., 2007), but in zebrafish, *sox2* was not involved in maintaining the pluripotency of stem cells, while played an important role in neuronal differentiation (Gou et al., 2018). With the advent of adult cell reprogramming techniques, the evaluation of pluripotency of induced pluripotent stem cells (iPS) has become particularly important (Stadtfeld and Hochedlinger, 2010; Feng et al., 2013). In the past decade, the research of iPSCs has mainly focused on mammals, but few attempts have been made in non-mammals (Takahashi and Yamanaka, 2006; Liao et al., 2009; Wu et al., 2009; Ben-Nun et al., 2011; Fuet and Pain, 2017; Pei et al., 2017; Pessoa et al., 2019). One of the challenges is the lack of suitable molecular markers to monitor the hypothetical pluripotency of iPS cells in non-mammalian (Pessoa et al., 2019; Liu et al., 2020).

In this study, we conducted a literature search on the list of genes which were reported relating to pluripotency, and screened out candidate genes. By examining their expression patterns in early fish embryos and the pluripotency of induced pluripotent stem cells in fish, we determined that three genes are suitable for identifying the pluripotency of induced pluripotent stem cells in fish. Our study is a step in the understanding of pluripotent markers of induced pluripotent stem cells in fish, and these genes are important tools for promoting research on the induction of fish pluripotent stem cells.

## Materials and Methods

### Fish

All the experiments were performed in strict accordance with the recommendations in the Guidelines for the Care and Use of Laboratory Animals of the National Advisory Committee for Laboratory Animal Research in China, and were approved by the Animal Care Committee of Hunan Normal University.

Zebrafish and crucian carp were maintained at the State Key Laboratory of Developmental Biology of Freshwater Fish, College of Life Sciences, Hunan Normal University. The embryos of zebrafish and crucian carp were collected at the stages of 256-cell, High, Oblong, Sphere, Dome, 30% epiboly, 50% epiboly, 75% epiboly, 3-somite, hatching, respectively. Under aseptic conditions, tissues (included liver, kidney, gut, skin, brain, heart, ovary, testis, etc.) were dissected from zebrafish (ZF) and crucian carp (CC), respectively.

### Generation of Induced Pluripotent Stem Cells

The induction of pluripotent stem cell from zebrafish fibroblasts was as previously described (Peng et al., 2019). For crucian carp, refered to our previous study (Zhou et al., 2016), the primary cells were isolated and prepared from caudal fin of 3 month old crucian carp and cultured in 3.5 cm culture dish. Then, trypsinized the fibroblasts (passage 5) and seeded at a density of 30,000–50,000 cells per well of a six well plate or 300,000 cells per 100 mm dish. We generated iPS-like cells from caudal fin fibroblasts of crucian carp with pure chemical reprogramming method (data unpublished). The iPS-like cells were cultured in fish stem cells culture medium, which was composed of DMEM supplemented with 7.5% FBS, 2.5% common carp serum, 0.1 ml of common carp fish embryo extract (100 embryos/mL), 1 mM sodium pyruvate, 0.1% 2-ME, 1 mM nonessential amino acids, 100 U/mL penicillin, 100 μg/ml streptomycin, 10 ng/ml bFGF, 0.5 μM ALK5 inhibitor, 0.5 μM MEK inhibitor, 3 μM GSK3*β* inhibitor, and 1000 U/mL leukemia inhibitory factor (For details, see Peng et al., 2019).

### Reverse Transcription PCR and Quantitative Real-Time PCR

Total RNAs were isolated from embryos and tissues using the Trizol Reagent (Takara, No.108-95-2) following the manufacturer’s protocol. And cDNAs were synthesized (Complementary Deoxyribonucleic acid) using Prime Script RT reagent Kit with gDNA Eraser (Takara Cat: RR047A). Reverse transcription PCR(RT-PCR) analysis was carried out with Taq DNA polymerase (TIANGEN). Quantitative real-time PCR (qRT-PCR) was conducted with SYBR Premix Ex Taq (bimake Cat: B21702) on an Applied Biosystems^®^ 7,500 Real-Time PCRSystem.The2-ΔΔCt method was used to analyze data. The primers were as follow Supplementary Material. For each sample, qRT-PCR analysis was conducted three times.

### Histological Sample Preparation

Tissues were surgically excised from fish under aseptic conditions, and fixed in 4% formaldehyde. After dehydrated with alcohol, the tissues were embedded in paraffin. Sections were cut at 5,6 μm, and some of them were stained with haematoxyl in and eosin, according to procedures described in previous study (Zhou et al., 2016).

### *In Situ* Hybridization

Perform experimental operations on prepared paraffin sections (Advanced Cell Diagnostics RNAscope**®** 2.0 HD Detection Kit) following the manufacturer’s protocol. Single-molecule *in situ* hybridization was performed using Advanced Cell Diagnostics RNAscope**®** 2.0 HD Detection Kit. Briefly, with 4% formalin-fixed and parafin-embedded, gonads tissues were sectioned at 5 µm. Tissues were deparafinized, dehydrated and treated with peroxidase block for 10 min at room temperature, then boiled in a pretreatment solution for 15 min and proteinase K treatment for 30 min at 40°C. Probes were hybridized for 2 h at 40°C, followed by a series of signal amplification (Amp) and washing steps. For RNA detection, incubation with the different amplifier solutions was performed in a water bath at 40°C. The pre-amplifier (2 nmol/L) was in hybridization buffer 2 (20% formamide, 5× SSC, 0.3% lithium dodecyl sulfate, 10% dextran sulfate, blocking reagents). The amplifier (2 nmol/L) was in hybridization buffer 2. The label probe (2 nmol/L) was in hybridization buffer 3 (5× SSC, 0.3% lithium dodecyl sulfate, blocking reagents) (Wang et al., 2012). After each hybridization step, the embryos were washed three times with 0.2× SSCT for 15 min (Wang et al., 2012). The sections were then incubated with DAPI ready-to-use solution (Advanced Cell Diagnostics) O/N at 24°C with slow agitation. Prior to imaging, embryos were rinsed in 0.01% PBT, mounted in 1% low melting point agarose (LMP) and imaged in 1× PBS solution (Gross-Thebing et al., 2014). After visualizing the fluorescent label, mount the slide and observe under a laser confocal scanning microscope (Olympus, FV10, Japan).

### Immunofluorescence

Cells were fixed in 4% paraformaldehyde for 30 min at 25°C and blocked with 2% BSA for 1 h. Primary antibodies of the following markers were used: Oct4 antibody (1:500; GeneTex Cat: GTX54240 United States), Nanog antibody (1:500; Cat. No. H0217; Santa Cruz, United States), Tdgf1 antibody (1:500; Cat. No. ab108391; abcam, United States). The fluorescently labeled secondary antibodies were anti-rabbit IgG for the antibodies of anti-Oct4 and anti-Tdgf1 (1:1000, invitrogen Cat: A11035, United States). The immunofluorescence images were observed and recorded under laser confocal scanning microscope (Olympus, FV10, Japan).

## Results

### Screening of Candidate Genes for Pluripotency Markers

**FIGURE 1:**
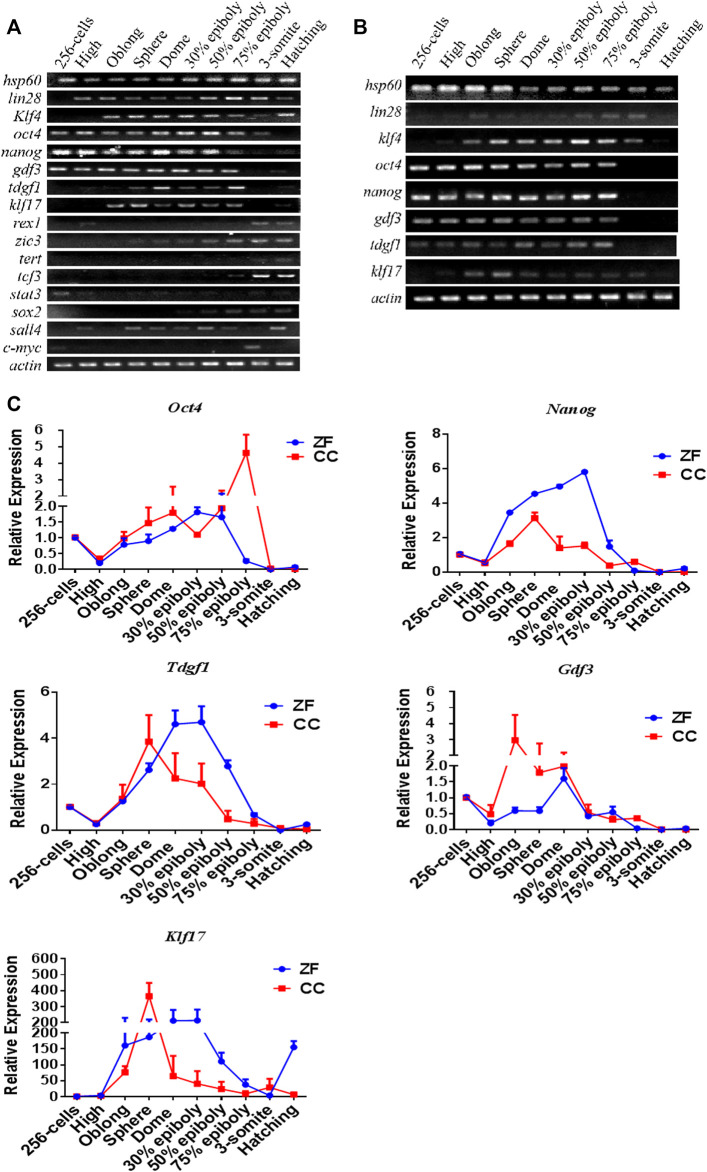
RT-PCR and qRT-PCR analysis of candidate pluripotency genes in 10 stages of em[bryonic development of Zebrafish (ZF) and Crucian carp (CC). **(A)** RT-PCR analysis of 16 pluripotency-related genes in 10 stages of embryonic development of zebrafish. **(B)** RT-PCR analysis of eight pluripotency-related genes in 10 stages of embryonic development of crucian carp. **(C)** qRT-PCR analysis for candidate pluripotency genes (*Nanog, Oct4, Tdgf1, Gdf3* and *Klf17*) through the 10 stages of ZF and CC embryos. It was clearly showed that these four genes were seemed to be mainly expressed in the pluripotent cells of undifferentiated embryos, but the expression was significantly decreased or even lost after embryonic cell differentiation. For each gene detected, the △CT value was calculated from the average CT value of the independent housekeeping gene *β*-actin. For each sample, At least three independent experiments were done for these results.

We collected the reported pluripotency gene data in mammals and identified 16 homologous genes in zebrafish through BLAST search in the public database (Table 1). We selected embryos from 10 critical development stages from cleavage to hatching and detected the expression pattern of pluripotent markers. RT-PCR analysis showed that the expression patterns of these 16 genes could be divided into three types in zebrafish. The first group maintained high expression throughout the embryonic stage, such as *Lin28*, *Hsp60* and *Klf4*. The expression of the second type was higher in the early stage of embryonic development, but it was significantly down-regulated after entering the somatic differentiation, such as *Oct4*, *Nanog*, *Gdf3*, *Klf17,* and *Tdgf1*. The third group included *Zic3, Sox2, Stat3, SALL4, Rex1, Tert, Tcf3,* and *C-myc*, whose expression levels were low throughout the embryonic stage, or only in the late embryonic stage (Figure 1A).

**TABLE 1:** Listed of candidate pluripotency genes.

Genes	References
*oct4*	Niwa et al. (2000); Takahashi and Yamanaka, (2006); Thomson et al. (2011); Sneha et al. (2019)
*sox2*	Avilion et al. (2003); Takahashi and Yamanaka, (2006); Sneha et al. (2019)
*c-myc*	Baudino et al., 2002; Adhikary and Eilers, 2005; Takahashi and Yamanaka, (2006)
*klf4*	Takahashi and Yamanaka, (2006)
*sall4*	Elling et al., 2006; Wu et al., 2014; Tatetsu et al. (2016)
*stat3*	Takeda et al. (1997)
*tcf3 *	Cole et al., 2008; Wang et al. (2011)
*tert*	Rao et al. (2011)
*gdf3*	Chen et al., 2006; Levine and Brivanlou, 2006; Pelliccia et al. (2017)
*nanog*	Chambers et al., 2003; Sneha et al. (2019)
*klf17*	Kotkamp et al. (2014)
*lin28*	Viswanathan et al. (2008); Grieco et al. (2013)
*zic3*	Lim et al. (2007)
*hsp60*	Gupta, (1995)
*rex1*	Hosler et al. (1989); Shi et al. (2006); Assadollahi et al. (2019)
*tdgf1*	Salomon et al. (1999); Scognamiglio et al. (1999); Bianco et al. (2009); Azizi et al. (2019)

The metaphase of fish blastocysts is a critical stage of pluripotency, which allows ES cells to be derived from embryos (Li et al., 2011; Ho et al., 2014; Hong et al., 2014). In crucian carp, the five genes of the second type expression pattern (*Oct4, Nanog, Gdf3, Tdgf1* and *KLf17*) was similar to that of zebrafish (Figure 1B). Therefore, we further verified the expression trend of these genes in embryonic development by qRT-PCR. As shown in Figure 1C, expression pattern of these five genes was similar in zebrafish and crucian carp. The expression of these genes was significantly up-regulated at the blastocyst stage, decreased gradually after entering the gastrula stage, reached the lowest point at the stage of somite differentiation. *Klf17* was shown some different expression patterns, such as non-maternal expression, and the RNA level of zebrafish enhanced after somite formation stage (Figure 1C). The results showed that *Oct4, Nanog, Gdf3*, and *Tdgf1* were seemed to be mainly expressed in the pluripotent cells of undifferentiated embryos, but the expression was significantly decreased or even lost after embryonic cell differentiation.

### Expression Pattern of Candidate Pluripotent Genes in Adult Tissues

**FIGURE 2:**
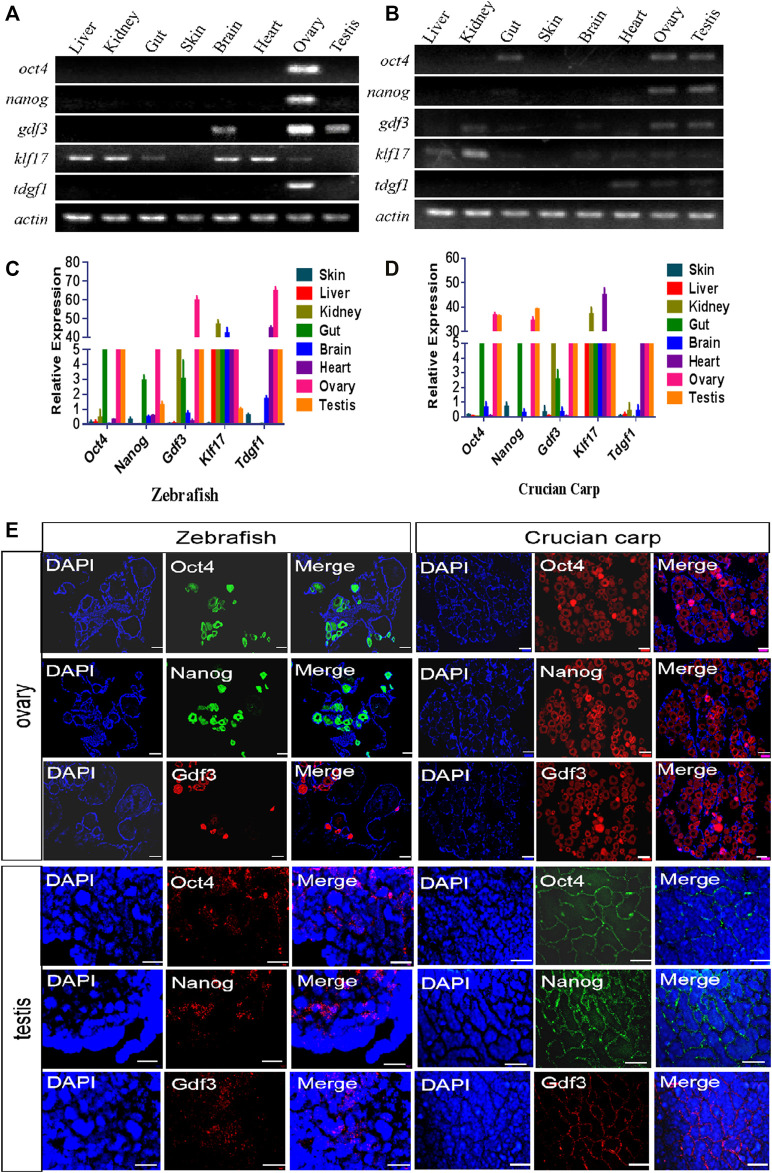
Expression pattern of candidate pluripotent genes in adult tissues. **(A,B)** RT-PCR analysis of the expression of five pluripotent genes in eight tissues of zebrafish **(A)** and crucian carp **(B)**. It was clearly showed that all of these genes were no expressed in skin tissue, but mainly expressed in ovary and testis, and some genes such as *Klf17* were widely expressed in the most of tissues. **(C,D)** qRT-PCR analysis of the expression of five pluripotent genes in eight tissues of zebrafish **(C)** and crucian carp **(D)**. The results showed that the expression patterns of these genes in different tissues of zebrafish and crucian carp were similar. *Oct4, Nanog* and *Gdf3* were mainly expressed in ovary, testis and gut, and *Gdf3* was also expressed in kidney. *Klf17* was expressed in all eight tissues. *Tdgf1* was mainly expressed in ovary, testis and heart. **(E)** Analysis of mRNA levels in the ovaries and testis of zebrafish and crucian carp after fluorescence *in situ* hybridization with Oct4, Nanog and Gdf3 probes. It was clearly showed that these three genes were strongly expressed in early oocytes and the outermost spermatogonia of the seminal lobules. The gonads were co-stained with DAPI. At least three independent experiments were done for these results. The scale bars in ovary were equal to 200 μm, while 50 μm in testis.

The expression patterns of above five genes in eight adult tissues of zebrafish and crucian carp (liver, kidney, intestine, skin, brain, heart, ovary and testis) were detected by RT-PCR (Figures 2A,B). The results showed that all of these genes were no expressed in skin tissue, but mainly expressed in ovary and testis, and some genes such as *Klf17* were widely expressed in the most of tissues. QRT-PCR results showed that the expression patterns of these genes in different tissues of zebrafish and crucian carp were similar. *Oct4, Nanog* and *Gdf3* were mainly expressed in ovary, testis and gut, and *Gdf3* was also expressed in kidney. *Klf17* was expressed in all eight tissues. *Tdgf1* was mainly expressed in ovary, testis and heart (Figures 2C,D).

We further selected *Oct4*, *Nanog* and *Gdf3* as representatives of pluripotent markers for fluorescence *in situ* hybridization of adult ovaries and testis. As shown in Figure 2E, these three genes were strongly expressed in early oocytes. In the testis, the three genes were expressed in the outermost spermatogonia of the seminal lobules, but not in the differentiated spermatogenic cells inside.

### Expression Pattern of Pluripotent Candidate Genes in iPS-Like Cells

**FIGURE 3:**
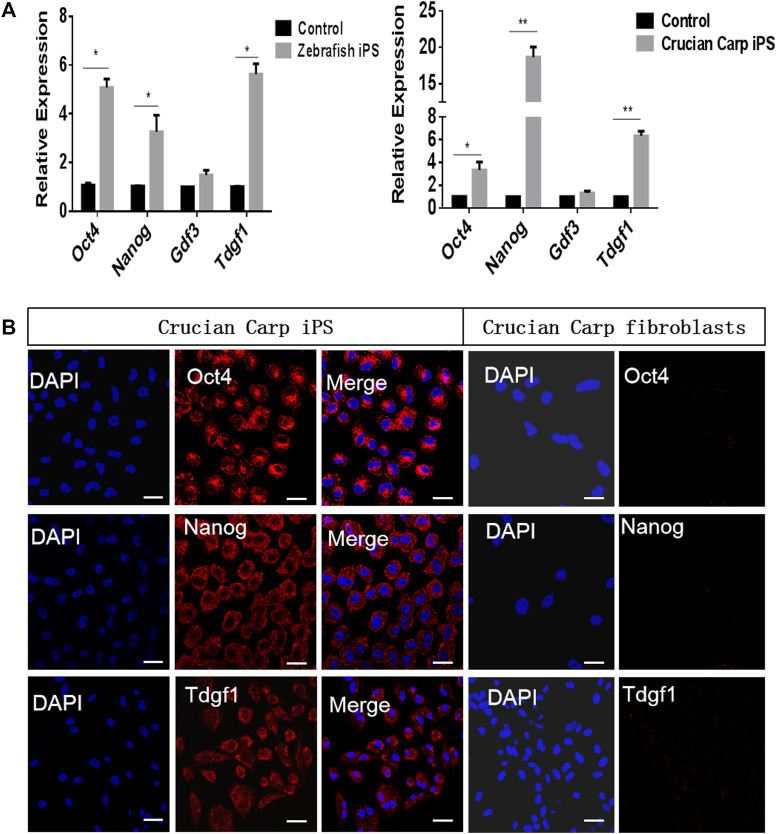
Expression pattern of pluripotent candidate genes in iPS-like cells from zebrafish and crucian carp. **(A)** qRT-PCR analyse of pluripotent candidate genes in iPS-like cells from zebrafish (left) and crucian carp (right). The fibroblasts from zebrafish and crucian as control, respectively. It was showed that the mRNA levels of *Oct4, Nanog,* and *Tdgf1* were higher in the iPS-like cells of zebrafish and crucian carp, while the expression level of *Gdf3* was lower. **(B)** Immunofluorescence staining of *Oct4, Nanog,* and *Tdgf1* iPS-like cells (passage 15) from crucian carp (Scale bars represent 20 μm). It was showed that the expression was positive in iPS-like cells, but not in fibroblasts. Data are shown as mean ± SD of values obtained from three independent experiments, and significant differences were evaluated using Student’s t test (*p < .005; **p < .001). The scale bars are equal to 20 μm.

The iPS-like cells from zebrafish and crucian carp were generated by chemical small molecules (Supplementary Material). The qRT-PCR results showed that the mRNA levels of *Oct4, Nanog,* and *Tdgf1* were higher in the iPS-like cells of zebrafish and crucian carp, while the expression level of *Gdf3* was lower (Figure 3A). Therefore, we chose *Oct4, Nanog* and *Tdgf1* as representatives, and detected the protein levels in the two kinds of iPS-like cells by immunofluorescence staining. The results showed that it was positive in the iPS-like cells, but not in the fibroblasts as the control group (Figure3B). Our results showed that *Oct4, Nanog* and *Tdgf1* could be used to label fish iPS-like cells.

## Discussion

In this study, we reported *Oct4, Nanog, Gdf3,* and *Tdgf1* were highly expressed in the short period of early embryonic development, but significantly down-regulated after differentiation. Moreover, three genes (*Oct4, Nanog,* and *Tdgf1*) have been verified in the detection of induced pluripotent stem cells in fish. Among them, *Oct4* and *Nanog* were commonly used marker genes in mammalian iPS cells (Ralston and Rossant, 2010), while *Tdgf1* seems to be a characteristic gene to identify the pluripotency status of fish iPS-like cells.

The gene *Oct4* was one of the important markers of cellular pluripotency in mammals and fish (Kellner and Kikyo, 2010; Onichtchouk, 2012; Liu et al., 2015; Onichtchouk, 2016). It plays a central role in maintaining the self-renewal and differentiation of embryonic stem cells into specific cell lines. In zebrafish, *Oct4* was continuously expressed from egg to late gastrula (Sanchez-Sanchez et al., 2011), and the expression level in blastocyst stage was higher than that in adult cells, which indicated that *Oct4* could maintain pluripotency (Robles et al., 2011). *Nanog* was a highly specifically expressed gene, mainly expressed in mammalian embryonic stem cells (Mitsui et al., 2003; Robles et al., 2011). Unlike mammals, *Nanog* could be expressed in the gonads of fish (Marandel et al., 2012). However, iPS cells could be produced when the *Nanog* gene in mice was replaced by the *Nanog* gene in zebrafish (Theunissen et al., 2011). The embryonic stem cells of herring with *Nanog* deletion had a tendency to differentiate, and their self-renewal ability would be destroyed to a great extent (Chambers et al., 2007). In mammals, *Tgdf1* (Cripto-1) is the downstream target gene of *Oct4* and *Nanog*, and is a potential pluripotent marker gene (Niemeyer et al., 1998; Watanabe et al., 2010). It was heterologous expressed in human embryonic stem cells and co-expressed with *Nanog* (Loh et al., 2006). It plays an important role in the early embryonic development and the regeneration and differentiation of stem cells (Chambers et al., 2007; Bianco et al., 2009). There were few studies on *Tgdf1* gene in fish, especially those related to pluripotency (Garland et al., 2019; Hoover et al., 2019). Our data showed that *Oct4*, *Nanog,* and *Tgdf1* were characteristic genes to identify the pluripotency status of fish iPS-like cells. As maternal factors, transcripts of these genes were uniformly distributed in the nucleus and cytoplasm of early oocytes, which were similar to the distribution of mRNA/protein of pluripotency-specific transcriptional factors in the early ovary of medaka (Liu et al., 2015). *Gdf3* is highly expressed in mammalian embryonic stem cells and control the differentiation of mouse and human embryonic stem cells. *Gdf3* plays a role in the formation of mesoderm and endoderm in the pre-development stage of gastrula (Levine and Brivanlou, 2006). In zebrafish, *Gdf3* is an essential cofactor of Nodal signaling during establishment of the embryonic axis (Bisgrove et al., 2017). Our data show that *Gdf3* is continuously expressed during the embryonic development of zebrafish and crucian carp, but its expression is low in iPS-like cells, so it is not suitable to be used as a marker of iPS-like cells.

Previous studies have shown that there are differences among iPS cell lines of different cell types and species (Martins-Taylor and Xu, 2010; Sanchez-Sanchez et al., 2011). In mammals, there are many established characteristics based on the pluripotency of embryonic stem cells, and iPS cells must meet these characteristics in order to be considered pluripotent (Smith et al., 2009). In addition to colony morphology, the characteristics of iPS cells was determined by the positive alkaline phosphatase (AKP) staining, the expression of pluripotent marker genes, formation of embryoid bodies and teratoma, and the potential to differentiate into germ cell-like cells *in vitro* (Smith et al., 2009; Marti et al., 2013). Fish species have been proved to be of great value in the study of development, evolution, environment and human diseases. Although, there are still many difficulties in establish a standard for pluripotency in reprogrammed fish cells. However, the induced pluripotent stem cell line in fish provides a useful model for the study of pluripotency in fish cell. And a better understanding of pluripotent marker genes in fish pluripotent stem cells also can promote the application of cell reprogramming in fish species.

## Data Availability

The original contributions presented in the study are included in the article/Supplementary Materials, further inquiries can be directed to the corresponding author.

## References

[B1] Adhikary S. Eilers M. (2005). Transcriptional Regulation and Transformation by Myc Proteins. Nat. Rev. Mol. Cel Biol 6 (8), 635–645. 10.1038/nrm1703 16064138

[B2] Assadollahi V. Hassanzadeh K. Abdi M. Alasvand M. Nasseri S. Fathi F. (2019). Effect of Embryo Cryopreservation on Derivation Efficiency, Pluripotency, and Differentiation Capacity of Mouse Embryonic Stem Cells. J. Cel Physiol 234 (12), 21962–21972. 10.1002/jcp.28759 31081207

[B3] Avilion A. A. Nicolis S. K. Pevny L. H. Perez L. Vivian N. Lovell-Badge R. (2003). Multipotent Cell Lineages in Early Mouse Development Depend on Sox2 Function. Genes Dev. 17 (1), 126–140. 10.1101/gad.224503 12514105 PMC195970

[B4] Azizi H. Asgari B. Skutella T. (2019). Pluripotency Potential of Embryonic Stem Cell-like Cells Derived from Mouse Testis. Cell J 21 (3), 281–289. 10.22074/cellj.2019.6068 31210434 PMC6582425

[B5] Barnes D. W. Parton A. Tomana M. Hwang J. H. Czechanski A. Fan L. (2008). “Stem Cells from Cartilaginous and Bony Fish, Stem Cell Culture., 343–367. 10.1016/s0091-679x(08)00016-2 18442656

[B6] Baudino T. A. McKay C. Pendeville-Samain H. Nilsson J. A. Maclean K. H. White E. L. (2002). C-myc Is Essential for Vasculogenesis and Angiogenesis during Development and Tumor Progression. Genes Dev. 16 (19), 2530–2543. 10.1101/gad.1024602 12368264 PMC187450

[B7] Bianco C. Cotten C. Lonardo E. Strizzi L. Baraty C. Mancino M. (2009). Cripto-1 Is Required for Hypoxia to Induce Cardiac Differentiation of Mouse Embryonic Stem Cells. Am. J. Pathol. 175 (5), 2146–2158. 10.2353/ajpath.2009.090218 19834060 PMC2774077

[B8] Bisgrove B. W. Su Y. C. Yost H. J. (2017). Maternal Gdf3 Is an Obligatory Cofactor in Nodal Signaling for Embryonic axis Formation in Zebrafish. Elife 6. 10.7554/eLife.28534 PMC5745076 29140249

[B9] Brambrink T. Foreman R. Welstead G. G. Lengner C. J. Wernig M. Suh H. (2008). Sequential Expression of Pluripotency Markers during Direct Reprogramming of Mouse Somatic Cells. Cell Stem Cell 2 (2), 151–159. 10.1016/j.stem.2008.01.004 18371436 PMC2276627

[B10] Chambers I. Colby D. Robertson M. Nichols J. Lee S. Tweedie S. (2003). Functional Expression Cloning of Nanog, a Pluripotency Sustaining Factor in Embryonic Stem Cells. Cell 113 (5), 643–655. 10.1016/S0092-8674(03)00392-1 12787505

[B11] Chambers I. Silva J. Colby D. Nichols J. Nijmeijer B. Robertson M. (2007). Nanog Safeguards Pluripotency and Mediates Germline Development. Nature 450 (7173), 1230–1234. 10.1038/nature06403 18097409

[B12] Chen C. Ware S. M. Sato A. Houston-Hawkins D. E. Habas R. Matzuk M. M. (2006). The Vg1-Related Protein Gdf3 Acts in a Nodal Signaling Pathway in the Pre-gastrulation Mouse Embryo. Development 133 (2), 319–329. 10.1242/dev.02210 16368929

[B13] Christen B. Robles V. Raya M. Paramonov I. Belmonte J. C. I. (2010). Regeneration and Reprogramming Compared. BMC Biol. 8, 5. 10.1186/1741-7007-8-5 20089153 PMC2826312

[B14] Cole M. F. Johnstone S. E. Newman J. J. Kagey M. H. Young R. A. (2008). Tcf3 Is an Integral Component of the Core Regulatory Circuitry of Embryonic Stem Cells. Genes Dev. 22 (6), 746–755. 10.1101/gad.1642408 18347094 PMC2275428

[B15] Elling U. Klasen C. Eisenberger T. Anlag K. Treier M. (2006). Murine Inner Cell Mass-Derived Lineages Depend on Sall4 Function. Proc. Natl. Acad. Sci. 103 (44), 16319–16324. 10.1073/pnas.0607884103 17060609 PMC1637580

[B16] Evans M. J. Kaufman M. H. (1981). Establishment in Culture of Pluripotential Cells from Mouse Embryos. Nature 292 (5819), 154–156. 10.1038/292154a0 7242681

[B17] Fan Z. Liu L. Huang X. Zhao Y. Zhou L. Wang D. (2017). Establishment and Growth Responses of Nile tilapia Embryonic Stem-like Cell Lines under Feeder-free Condition. Develop. Growth Differ. 59 (2), 83–93. 10.1111/dgd.12341 28230233

[B18] Feng C. Jia Y.-D. Zhao X.-Y. (2013). Pluripotency of Induced Pluripotent Stem Cells. Genomics, Proteomics & Bioinformatics 11 (5), 299–303. 10.1016/j.gpb.2013.08.003 PMC4357825 24100275

[B19] Friedrich Ben-Nun I. Montague S. C. Houck M. L. Tran H. T. Garitaonandia I. Leonardo T. R. (2011). Induced Pluripotent Stem Cells from Highly Endangered Species. Nat. Methods 8 (10), 829–831. 10.1038/nmeth.1706 21892153

[B20] Fuet A. Pain B. (2017). Chicken Induced Pluripotent Stem Cells: Establishment and Characterization. Methods Mol. Biol. 1650, 211–228. 10.1007/978-1-4939-7216-6_14 28809024

[B21] Garland M. A. Sengupta S. Mathew L. K. Truong L. de Jong E. Piersma A. H. (2019). Glucocorticoid Receptor-dependent Induction of Cripto-1 (One-eyed Pinhead) Inhibits Zebrafish Caudal Fin Regeneration. Toxicol. Rep. 6, 529–537. 10.1016/j.toxrep.2019.05.013 31249786 PMC6584771

[B22] Gou Y. Vemaraju S. Sweet E. M. Kwon H.-J. Riley B. B. (2018). sox2 and Sox3 Play Unique Roles in Development of Hair Cells and Neurons in the Zebrafish Inner Ear. Develop. Biol. 435 (1), 73–83. 10.1016/j.ydbio.2018.01.010 29355523 PMC5818298

[B23] Grieco A. Rzeczkowska P. Alm C. Palmert M. R. (2013). Investigation of Peripubertal Expression of Lin28a and Lin28b in C57bl/6 Female Mice. Mol. Cell Endocrinol. 365 (2), 241–248. 10.1016/j.mce.2012.10.025 23138112 PMC3529789

[B24] Gupta R. S. (1995). Evolution of the Chaperonin Families (HSP60, HSP 10 and TCP-1) of Proteins and the Origin of Eukaryotic Cells. Mol. Microbiol. 15 (1), 1–11. 10.1111/j.1365-2958.1995.tb02216.x 7752884

[B25] Hanna J. H. Saha K. Jaenisch R. (2010). Pluripotency and Cellular Reprogramming: Facts, Hypotheses, Unresolved Issues. Cell 143 (4), 508–525. 10.1016/j.cell.2010.10.008 21074044 PMC3032267

[B26] Ho S. Y. Goh C. W. P. Gan J. Y. Lee Y. S. Lam M. K. K. Hong N. (2014). Derivation and Long-Term Culture of an Embryonic Stem Cell-like Line from Zebrafish Blastomeres under Feeder-free Condition. Zebrafish 11 (5), 407–420. 10.1089/zeb.2013.0879 24967707 PMC4172385

[B27] Hoffman J. A. Merrill B. J. (2007). New and Renewed Perspectives on Embryonic Stem Cell Pluripotency. Front. Biosci. 12, 3321–3332. 10.2741/2315 17485302

[B28] Hong N. Schartl M. Hong Y. (2014). Derivation of Stable Zebrafish ES-like Cells in Feeder-free Culture. Cell Tissue Res 357 (3), 623–632. 10.1007/s00441-014-1882-0 24850275

[B29] Hong Y. Winkler C. Schartl M. (1996). Pluripotency and Differentiation of Embryonic Stem Cell Lines from the Medakafish (*Oryzias latipes*). Mech. Develop. 60 (1), 33–44. 10.1016/s0925-4773(96)00596-5 9025059

[B30] Hong Y. Winkler C. Schartl M. (1998). Production of Medakafish Chimeras from a Stable Embryonic Stem Cell Line. Proc. Natl. Acad. Sci. 95 (7), 3679–3684. 10.1073/pnas.95.7.3679 9520425 PMC19895

[B31] Hoover M. Runa F. Booker E. Diedrich J. K. Duell E. Williams B. (2019). Identification of Myosin II as a Cripto Binding Protein and Regulator of Cripto Function in Stem Cells and Tissue Regeneration. Biochem. Biophysical Res. Commun. 509 (1), 69–75. 10.1016/j.bbrc.2018.12.059 PMC6855394 30579599

[B32] Hosler B. A. LaRosa G. J. Grippo J. F. Gudas L. J. (1989). Expression of rex-1, a Gene Containing Zinc finger Motifs, Is Rapidly Reduced by Retinoic Acid in F9 Teratocarcinoma Cells. Mol. Cel. Biol. 9 (12), 5623–5629. 10.1128/MCB.9.12.5623 PMC363733 2511439

[B33] Kellner S. Kikyo N. (2010). Transcriptional Regulation of the Oct4 Gene, a Master Gene for Pluripotency. Histol. Histopathol 25 (3), 405–412. 10.14670/HH-25.405 20054811 PMC3418322

[B34] Kotkamp K. Mössner R. Allen A. Onichtchouk D. Driever W. (2014). A Pou5f1/oct4 Dependent Klf2a, Klf2b, and Klf17 Regulatory Sub-network Contributes to Evl and Ectoderm Development during Zebrafish Embryogenesis. Develop. Biol. 385 (2), 433–447. 10.1016/j.ydbio.2013.10.025 24211655

[B35] Kumar A. Tripathi G. Vimal B. Bedekar M. K. Kumar A. P. (2020). Expression Study of Pluripotency Marker Genes in Gold Fish, Carassius Auratrus. Int.J.Curr.Microbiol.App.Sci 9 (4), 639–649. 10.20546/ijcmas.2020.904.078

[B36] Levine A. J. Brivanlou A. H. (2006). GDF3, a BMP Inhibitor, Regulates Cell Fate in Stem Cells and Early Embryos. Development 133 (2), 209–216. 10.1242/dev.02192 16339188

[B37] Li M. Belmonte J. C. I. (2017). Ground Rules of the Pluripotency Gene Regulatory Network. Nat. Rev. Genet. 18 (3), 180–191. 10.1038/nrg.2016.156 28045100

[B38] Li Q. V. Rosen B. P. Huangfu D. (2020). Decoding Pluripotency: Genetic Screens to Interrogate the Acquisition, Maintenance, and Exit of Pluripotency. Wires Syst. Biol. Med. 12 (1), e1464. 10.1002/wsbm.1464 PMC6898739 31407519

[B39] Li Z. Bhat N. Manali D. Wang D. Hong N. Yi M. (2011). Medaka Cleavage Embryos Are Capable of Generating ES-like Cell Cultures. Int. J. Biol. Sci. 7 (4), 418–425. 10.7150/ijbs.7.418 21547059 PMC3088284

[B40] Liao J. Cui C. Chen S. Ren J. Chen J. Gao Y. (2009). Generation of Induced Pluripotent Stem Cell Lines from Adult Rat Cells. Cell Stem Cell 4 (1), 11–15. 10.1016/j.stem.2008.11.013 19097959

[B41] Lim L. S. Loh Y.-H. Zhang W. Li Y. Chen X. Wang Y. (2007). Zic3 Is Required for Maintenance of Pluripotency in Embryonic Stem Cells. MBoC 18 (4), 1348–1358. 10.1091/mbc.e06-07-0624 17267691 PMC1838990

[B42] Liu G. David B. T. Trawczynski M. Fessler R. G. (2020). Advances in Pluripotent Stem Cells: History, Mechanisms, Technologies, and Applications. Stem Cel Rev Rep 16 (1), 3–32. 10.1007/s12015-019-09935-x PMC6987053 31760627

[B43] Liu R. Li M. Li Z. Hong N. Xu H. Hong Y. (2015). Medaka Oct4 Is Essential for Pluripotency in Blastula Formation and ES Cell Derivation. Stem Cel Rev Rep 11 (1), 11–23. 10.1007/s12015-014-9523-2 25142379

[B44] Loh Y.-H. Wu Q. Chew J.-L. Vega V. B. Zhang W. Chen X. (2006). The Oct4 and Nanog Transcription Network Regulates Pluripotency in Mouse Embryonic Stem Cells. Nat. Genet. 38 (4), 431–440. 10.1038/ng1760 16518401

[B45] Marandel L. Labbe C. Bobe J. Le Bail P.-Y. (2012). Nanog 5′-upstream Sequence, DNA Methylation, and Expression in Gametes and Early Embryo Reveal Striking Differences between Teleosts and Mammals. Gene 492 (1), 130–137. 10.1016/j.gene.2011.10.037 22037485

[B46] Martí M. Mulero L. Pardo C. Morera C. Carrió M. Laricchia-Robbio L. (2013). Characterization of Pluripotent Stem Cells. Nat. Protoc. 8 (2), 223–253. 10.1038/nprot.2012.154 23306458

[B47] Martins-Taylor K. Xu R.-H. (2009). Determinants of Pluripotency: from Avian, Rodents, to Primates. J. Cel. Biochem. 109 (1), a–n. 10.1002/jcb.22402 19937733

[B48] Mitsui K. Tokuzawa Y. Itoh H. Segawa K. Murakami M. Takahashi K. (2003). The Homeoprotein Nanog Is Required for Maintenance of Pluripotency in Mouse Epiblast and Es Cells. Cell 113 (5), 631–642. 10.1016/S0092-8674(03)00393-3 12787504

[B49] Ng H.-H. Surani M. A. (2011). The Transcriptional and Signalling Networks of Pluripotency. Nat. Cel Biol 13 (5), 490–496. 10.1038/ncb0511-490 21540844

[B50] Niemeyer C. C. Persico M. G. Adamson E. D. (1998). Cripto: Roles in Mammary Cell Growth, Survival, Differentiation and Transformation. Cell Death Differ 5 (5), 440–449. 10.1038/sj.cdd.4400368 10200494

[B51] Niwa H. Miyazaki J.-i. Smith A. G. (2000). Quantitative Expression of Oct-3/4 Defines Differentiation, Dedifferentiation or Self-Renewal of Es Cells. Nat. Genet. 24 (4), 372–376. 10.1038/74199 10742100

[B52] Notarianni E. Laurie S. Moor R. M. Evans M. J. (1990). Maintenance and Differentiation in Culture of Pluripotential Embryonic Cell Lines from Pig Blastocysts. J. Reprod. Fertil. Suppl. 41, 51–56. 2213715

[B53] Onichtchouk D. (2016). Evolution and Functions of Oct4 Homologs in Non-mammalian Vertebrates. Biochim. Biophys. Acta (Bba) - Gene Regul. Mech. 1859 (6), 770–779. 10.1016/j.bbagrm.2016.03.013 27058398

[B54] Onichtchouk D. (2012). Pou5f1/oct4 in Pluripotency Control: Insights from Zebrafish. Genesis 50 (2), 75–85. 10.1002/dvg.20800 21913309

[B55] Paranjpe S. S. Veenstra G. J. C. (2015). Establishing Pluripotency in Early Development. Biochim. Biophys. Acta (Bba) - Gene Regul. Mech. 1849 (6), 626–636. 10.1016/j.bbagrm.2015.03.006 PMC4437833 25857441

[B56] Pei H. Fu H.-Y. Hirai H. Cho D. S. O'Brien T. D. Dutton J. (2017). Generation of Induced Pluripotent Stem Cells from Chinese Hamster Embryonic Fibroblasts. Stem Cel Res. 21, 132–136. 10.1016/j.scr.2017.04.010 28677528

[B57] Pelliccia J. L. Jindal G. A. Burdine R. D. (2017). Gdf3 Is Required for Robust Nodal Signaling during Germ Layer Formation and Left-Right Patterning. Elife 6, e28635. 10.7554/eLife.28635 29140250 PMC5745080

[B58] Peng L. Zhou Y. Xu W. Jiang M. Li H. Long M. (2019). Generation of Stable Induced Pluripotent Stem-like Cells from Adult Zebra Fish Fibroblasts. Int. J. Biol. Sci. 15 (11), 2340–2349. 10.7150/ijbs.34010 31595152 PMC6775306

[B59] Pessôa L. V. d. F. Bressan F. F. Freude K. K. (2019). Induced Pluripotent Stem Cells throughout the Animal Kingdom: Availability and Applications. Wjsc 11 (8), 491–505. 10.4252/wjsc.v11.i8.491 31523369 PMC6716087

[B60] Ralston A. Rossant J. (2010). The Genetics of Induced Pluripotency. Reproduction 139 (1), 35–44. 10.1530/REP-09-0024 19605512

[B61] Rao F. Wang T. Li M. Li Z. Hong N. Zhao H. (2011). Medaka Tert Produces Multiple Variants with Differential Expression during Differentiation *In Vitro* and *In Vivo* . Int. J. Biol. Sci. 7 (4), 426–439. 10.7150/ijbs.7.426 21547060 PMC3088285

[B62] Robles V. Martí M. Belmonte J. C. I. (2011). Study of Pluripotency Markers in Zebrafish Embryos and Transient Embryonic Stem Cell Cultures. Zebrafish 8 (2), 57–63. 10.1089/zeb.2010.0684 21563922 PMC3472678

[B63] Salomon D. S. Bianco C. De Santis M. (1999). Cripto: a Novel Epidermal Growth Factor (EGF)-related Peptide in Mammary Gland Development and Neoplasia. Bioessays 21 (1), 61–70. 10.1002/(SICI)1521-1878(199901)21:13.0.CO;2-H10.1002/(sici)1521-1878(199901)21:1<61:aid-bies8>3.0.co;2-h 10070255

[B64] Sánchez-Sánchez A. V. Camp E. Mullor J. L. (2011). Fishing Pluripotency Mechanisms *In Vivo* . Int. J. Biol. Sci. 7 (4), 410–417. 10.7150/ijbs.7.410 21547058 PMC3088283

[B65] Scognamiglio B. Baldassarre G. Cassano C. Tucci M. Montuori N. Dono R. (1999). Assignment of Human Teratocarcinoma Derived Growth Factor (Tdgf) Sequences to Chromosomes 2q37, 3q22, 6p25 and 19q13.1. Cytogenet. Genome Res. 84 (3-4), 220–224. 10.1159/000015263 10393436

[B66] Shamblott M. J. Axelman J. Wang S. Bugg E. M. Littlefield J. W. Donovan P. J. (1998). Derivation of Pluripotent Stem Cells from Cultured Human Primordial Germ Cells. Proc. Natl. Acad. Sci. 95 (23), 13726–13731. 10.2307/4675410.1073/pnas.95.23.13726 9811868 PMC24887

[B67] Shi W. Wang H. Pan G. Geng Y. Guo Y. Pei D. (2006). Regulation of the Pluripotency Marker rex-1 by Nanog and Sox2. J. Biol. Chem. 281 (33), 23319–23325. 10.1074/jbc.M601811200 16714766

[B68] Smith K. P. Luong M. X. Stein G. S. (2009). Pluripotency: toward a Gold Standard for Human ES and iPS Cells. J. Cel. Physiol. 220 (1), 21–29. 10.1002/jcp.21681 19326392

[B69] Sneha S. Nagare R. P. Manasa P. Vasudevan S. Shabna A. Ganesan T. S. (2019). Analysis of Human Stem Cell Transcription Factors. Cell Reprogramming 21 (4), 171–180. 10.1089/cell.2019.0005 31298562

[B70] Stadtfeld M. Hochedlinger K. (2010). Induced Pluripotency: History, Mechanisms, and Applications. Genes Dev. 24 (20), 2239–2263. 10.1101/gad.1963910 20952534 PMC2956203

[B71] Surani M. A. Hayashi K. Hajkova P. (2007). Genetic and Epigenetic Regulators of Pluripotency. Cell 128 (4), 747–762. 10.1016/j.cell.2007.02.010 17320511

[B72] Takahashi K. Yamanaka S. (2006). Induction of Pluripotent Stem Cells from Mouse Embryonic and Adult Fibroblast Cultures by Defined Factors. Cell 126 (4), 663–676. 10.1016/j.cell.2006.07.024 16904174

[B73] Takeda K. Noguchi K. Shi W. Tanaka T. Matsumoto M. Yoshida N. (1997). Targeted Disruption of the Mouse Stat3 Gene Leads to Early Embryonic Lethality. Proc. Natl. Acad. Sci. 94 (8), 3801–3804. 10.1073/pnas.94.8.3801 9108058 PMC20521

[B74] Tatetsu H. Kong N. R. Chong G. Amabile G. Tenen D. G. Chai L. (2016). Sall4, the Missing Link between Stem Cells, Development and Cancer. Gene 584 (2), 111–119. 10.1016/j.gene.2016.02.019 26892498 PMC4823161

[B75] Theunissen T. W. Costa Y. Radzisheuskaya A. van Oosten A. L. Lavial F. Pain B. (2011). Reprogramming Capacity of Nanog Is Functionally Conserved in Vertebrates and Resides in a Unique Homeodomain. Development 138 (22), 4853–4865. 10.1242/dev.068775 22028025 PMC3201656

[B76] Thiagarajan R. D. Morey R. Laurent L. C. (2014). The Epigenome in Pluripotency and Differentiation. Epigenomics 6 (1), 121–137. 10.2217/epi.13.80 24579950

[B77] Thomson J. A. Itskovitz-Eldor J. Shapiro S. S. Waknitz M. A. Swiergiel J. J. Marshall V. S. (1998). Embryonic Stem Cell Lines Derived from Human Blastocysts. Science 282 (5391), 1145–1147. 10.1126/science.282.5391.1145 9804556

[B78] Thomson J. A. Kalishman J. Golos T. G. Durning M. Harris C. P. Becker R. A. (1995). Isolation of a Primate Embryonic Stem Cell Line. Proc. Natl. Acad. Sci. 92 (17), 7844–7848. 10.1073/pnas.92.17.7844 7544005 PMC41242

[B79] Thomson M. Liu S. J. Zou L.-N. Smith Z. Meissner A. Ramanathan S. (2011). Pluripotency Factors in Embryonic Stem Cells Regulate Differentiation into Germ Layers. Cell 145 (6), 875–889. 10.1016/j.cell.2011.05.017 21663792 PMC5603300

[B80] Viswanathan S. R. Daley G. Q. Gregory R. I. (2008). Selective Blockade of Microrna Processing by Lin28. Science 320 (5872), 97–100. 10.1126/science.1154040 18292307 PMC3368499

[B81] Wang D. Manali D. Wang T. Bhat N. Hong N. Li Z. (2011). Identification of Pluripotency Genes in the Fish Medaka. Int. J. Biol. Sci. 7 (4), 440–451. 10.7150/ijbs.7.440 21547061 PMC3088286

[B82] Watanabe K. Meyer M. J. Strizzi L. Lee J. M. Gonzales M. Bianco C. (2010). Cripto-1 Is a Cell Surface Marker for a Tumorigenic, Undifferentiated Subpopulation in Human Embryonal Carcinoma Cells. Stem Cells 28 (8), 1303–1314. 10.1002/stem.463 20549704 PMC3069615

[B83] Wu M. Yang F. Ren Z. Jiang Y. Ma Y. Chen C.-Y. (2014). Identification of the Nuclear Localization Signal of Sall4b, a Stem Cell Transcription Factor. Cell Cycle 13 (9), 1456–1462. 10.4161/cc.28418 24626181 PMC4050143

[B84] Wu Z. Chen J. Ren J. Bao L. Liao J. Cui C. (2009). Generation of Pig Induced Pluripotent Stem Cells with a Drug-Inducible System. J. Mol. Cel Biol 1 (1), 46–54. 10.1093/jmcb/mjp003 19502222

[B85] Xiao Y. Gao M. Gao L. Zhao Y. Hong Q. Li Z. (2016). Directed Differentiation of Zebrafish Pluripotent Embryonic Cells to Functional Cardiomyocytes. Stem Cel Rep. 7 (3), 370–382. 10.1016/j.stemcr.2016.07.020 PMC5032289 27569061

[B86] Yi M. Hong N. Hong Y. (2010). Derivation and Characterization of Haploid Embryonic Stem Cell Cultures in Medaka Fish. Nat. Protoc. 5 (8), 1418–1430. 10.1038/nprot.2010.104 20671725

[B87] Zhou Y. Wang M. Jiang M. Peng L. Wan C. Liu J. (2016). Autotetraploid Cell Line Induced by SP600125 from Crucian Carp and its Developmental Potentiality. Sci. Rep. 6, 21814. 10.1038/srep21814 26898354 PMC4761888

