# Supplementary material for: Defining the Pluripotent Marker Genes for Identification of Teleost Fish Cell Pluripotency During Reprogramming
Source: Front Genet. 2022 Feb 11;13:819682. doi: 10.3389/fgene.2022.819682 (PMC8874021; doi:10.3389/fgene.2022.819682)
Supplement: Supplementary file 1 [file DataSheet1.PDF]

Table S1. Primers used in RT-PCR and qRT-PCR

Name and Reference Sequence	Forward Primer	Reverse Primer
ZF-*oct4* NM_131112.1	GGCCGAAAACTCCGAGAA	TTGCAGAACCATACACGC
ZF-*nanog* JN 615142.1	TGTACCCGCAAGTGTCAGAG	TGAGCGGTAAAGTAGCCTG
ZF-*sox2* NM_213118.1	CGAGTCTAGTTCGAGTCCGC	TGAGCGGTAAAGTAGCCTG
ZF-*klf4* NM_131723.1	CAGATGCGATGTTGCCTTC	GATGCCCGTACGTTTGATGC
ZF-*c-myc* EF 194871.1	CTGCGATGATGCGGACTACT	C*CAT*AGTACGAGATACACAG
ZF-*klf17* NM_131723.1	GGACATTTAGACAGATTGGAG	GAGTGTTCATCTCTGTCAGG
ZF-*lin28* NM_201091.1	CCAATCATCATGCCAAAGAAT G	TGGCTCATGTCCTCCTCTTC
ZF-*gdf3* NM_130948.1	GGCCGTCCACAGCTTTAAC	CTCGG*CAT*AATCTGACTCC
ZF-*tdgf1* NM_001328408.1	AGTCAGGATGTGAGGGGTC	ATGCACGTTCCCCCATTCTT
ZF-*tert* NM_001083866.1	AGGTCTTGGCGTTCACTCTG	CACCATGGAAACGGAAGGGA
ZF-*tcf3* NM_131094.1	CGAAGGCATCATGGGAAAC	CTGGACTGCGGTAAACCTG
ZF-rex1 NM_001126416.1	GAGAACACCAATGCCAATGC	TTCTCCTCTGTCCCGCTAAA
ZF-stat3 NM_131479.1	TCGAGGTTCACGCAAGTTCA	GCCTCCATTGCCACATCTCT
ZF-zic3 NM_001001950.2	ACATCCCAGCTCTCTCAGG	TGTCGGGTCTTCAGTGTTCG
ZF-hsp60 NM_181330.3	GATCGTGTCACTGATGCCCT	CATCTAGCAGTGCCGTCCTC
ZF-sall4 NM_001080609.1	CAAGCAGTCCAAACCACAG	AGAACTCAGGGGGCACTTC
ZF-actin NM_131031.2	AGCCGAAAGAGAAATTGTCC	GAAACGCTCATTGCCGAT
RCC-oct4 GCF_003368295.1	CTCAAACACAAGCGCATCA	TGTCCACGAACACCCGTTCA

RCC-nanog GCF_003368295.1	TGATTCAGAGGCCCATACG	AGATCAGTTCTCCCCCGTCA
RCC-klf4 GCF_003368295.1	AACCAGGCCAATGTGTCCAA	TCCCGAGGCACTTCTTGTTG
RCC-klf17 GCF_003368295.1	GGACATTTCGACAGGCTGGA	CCCGCGTAGTCGTTTACCAT
RCC-lin28 GCF_003368295.1	TCGTCCATCAGCAGAGCAAG	CTTTGGCGTGATGATTCGGC
RCC-gdf3 GCF_003368295.1	AGATTGCTCCGATGCCAGA	AATGATCCAGTCCTGCCAGC
RCC-tdgf1 GCF_003368295.1	ACCTGCTGTAAGAACGGTGG	TGAACCCATTCTCCGTGTGG
RCC-hsp60 GCF_003368295.1	CTGGCGGTGGAAGAAGTCAT	TAATAACACCCTTGCGGCCC
RCC-actin GCF_003368295.1	CATCTACGAGGGTTACGCCC	AATTTCCCTCTCGGCTGTGG

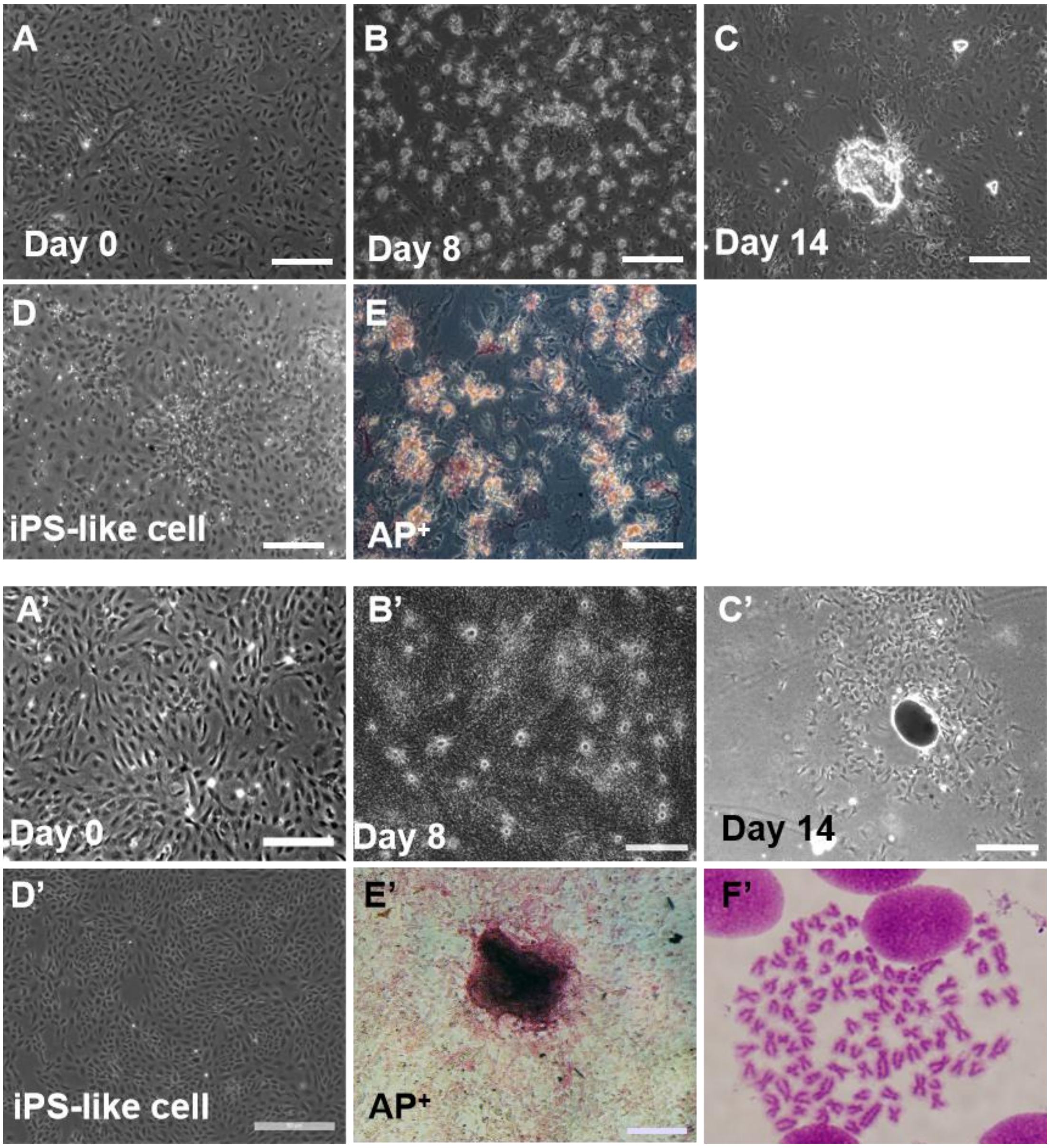

Figure S1 Generation of iPS cells from zebrafish and crucian carp fin fibroblasts.

(A, A') Morphology of the fibroblasts from zebrafish (A) and crucian carp (A') caudual fin.

(B, B') Morphology of the induced cell from zebrafish (B) and crucian carp (B') at day 8, aggregated cell clusters appeared.

(C, C') Morphology of the induced cell from zebrafish (C) and crucian carp (C') at day 14, a compact cell cluster appeared in the petri dish.

(D, D') Morphology of the iPS-like cells from zebrafish (D) and crucian carp (D'). After 10 courses in a fish stem cell culture medium, the stable ES cell-like

morphology was maintained.

(E, E’) Morphology of the clone from zebrafish (E) and crucian carp (E’), the alkaline phosphatase test showed positive

(F’) Karyotype analysis of iPS-like cells from crucian carp at passage 10, normal karyotype was maintained, The scale bars are equal to 50μm.
